# Decreasing aortic diameter and decreasing prevalence of infrarenal aortic aneurysms in a population-based screening programme

**DOI:** 10.1093/bjs/znaf156

**Published:** 2025-08-20

**Authors:** Antti Siika, Anton Axelsson, Nina Fattahi, Joy Roy, Daniel Öhman, Anneli Linné, Rebecka Hultgren

**Affiliations:** Department of Molecular Medicine and Surgery, Karolinska Institutet, Stockholm, Sweden; Department of Molecular Medicine and Surgery, Karolinska Institutet, Stockholm, Sweden; Department of Molecular Medicine and Surgery, Karolinska Institutet, Stockholm, Sweden; Department of Vascular Surgery, Karolinska Institutet and University Hospital, Stockholm, Sweden; Department of Molecular Medicine and Surgery, Karolinska Institutet, Stockholm, Sweden; Department of Vascular Surgery, Karolinska Institutet and University Hospital, Stockholm, Sweden; Department of Cancer Prevention and Screening, Regional Cancer Centre Stockholm-Gotland, Stockholm, Sweden; Department of Clinical Science and Education, Karolinska Institutet at Södersjukhuset, Stockholm, Sweden; Department of Surgery, Section of Vascular Surgery, Södersjukhuset, Stockholm, Sweden; Department of Molecular Medicine and Surgery, Karolinska Institutet, Stockholm, Sweden; Department of Vascular Surgery, Karolinska Institutet and University Hospital, Stockholm, Sweden

## Abstract

**Background:**

Temporal trends of infrarenal aortic diameters and their association with long-term mortality were explored in a population-based abdominal aortic aneurysm (AAA) screening programme. Additionally, changes in risk factor distribution and outcomes among the individuals with an AAA were analysed over the 14-year study period.

**Methods:**

In this population-based cohort study between 2010 and 2023, all 65-year-old men who had been invited to screening for AAA were studied (152 000). Aortic diameter and date of death were extracted from the regional screening database.

**Results:**

Some 117 120 men were examined, with reductions in mean(s.d.) aortic diameter (18.6(3.3) mm to 18.1(2.8) mm) and AAA prevalence (1.32% to 0.69%). The prevalence of small aortas (<17 mm) was 18.5% and that of subaneurysmal aortas (25–29 mm) was 1.1%. Initial aortic diameter showed a non-linear association with mortality (*P* < 0.001). The 5-year mortality was 3.8% for men with a normal aorta, 5.5% for men with a small aorta, 8.1% for men with a subaneurysmal aorta, and 9.5% for men with an AAA. The incidence of non-smoking men with an AAA remained constant, while a decline in the number of men with a smoking history was observed. Smoking cessation influenced timing of surgery and survival, with a 5-year mortality of 11.1% in current smokers *versus* 5.6% in non-smokers.

**Conclusion:**

There has been a decline in the prevalence of AAA and subaneurysmal aortas, and a slight rise in men with small aortas. Men with small or aneurysmatic aortas are at 1.5–2.5 times higher risk of mortality at 5 years compared with men with normal diameters. Smoking cessation halts the progression to AAA surgery and is associated with reduced mortality.

## Teaser

Infrarenal aortic diameter is decreasing together with the prevalence of AAA. Individuals with both small and large infrarenal aortic diameters, compared with normal, have an increased risk of all-cause mortality.

## Introduction

Abdominal aortic aneurysm (AAA) is an asymptomatic widening of the infrarenal aorta to ≥3 cm^1,2^. Common risk factors are male sex, increasing age, smoking, heredity, other arterial aneurysms, and other manifestations of cardiovascular disease (CVD)^1,3,4^. This multifactorial disease has life-threatening consequences; if rupture occurs, mortality is 100% if left untreated. There is clear evidence that a population-based screening programme is cost-effective with regard to minimizing the risk of premature aneurysm-related death; since early identification of patients with a small AAA is essential to minimize the risk of rupture^2,3,5^. Population-based screening is ongoing in the UK and in Sweden, and a modified targeted screening is performed in the USA (Medicare)^2,6,7^. An exploration of the quality of the Swedish national programme showed a high participation rate, but also a declining prevalence of AAA, and similar patterns are also reported in the UK^8,9^.

During the past two decades manifest shifts in factors contributing to CVD have been reported; a declining proportion of smoking men and an increased prescription of statins^10,11^. In Sweden, decreased smoking has been coupled with an increase in the use of smokeless tobacco (Swedish snus)^12^. Its association with the development of CVD, and particularly AAA, is rather uncertain^13^.

In addition to identifying individuals with an AAA, a screening programme can also stratify individuals at risk of developing an aortic aneurysm or other health conditions based on infrarenal aortic diameter. A growing area of interest is men diagnosed with a subaneurysmal aorta (often defined as an aortic diameter of 25–29 mm)^2^. An often overlooked surrogate marker of severe cardiovascular risk is a small aortic diameter^14–17^. A small aortic diameter is associated with atherosclerosis, but there is lack of knowledge of the contemporary mortality risk for these persons compared with men with normal aortic diameters.

The primary aim of this study was to explore temporal trends in infrarenal aortic diameters in the screening population and the association of aortic diameter with all-cause mortality. The secondary aim was to explore if the men diagnosed during the 14 years with an AAA had a changing set of risk factors and how these factors influenced aneurysm progression.

## Methods

Stockholm Region, formerly called Stockholm County, had a population of 2.46 million inhabitants in 2024 and has an area of 6500 km^2^. The healthcare services of Stockholm Region include those of Gotland Island Region with regard to vascular surgery and screening. Stockholm Region started a population-based, organized screening programme in August 2010, inviting all 65-year-old men to a one-time ultrasonographic screening, that has been described previously^18^. The ultrasonographic methodology used is the Leading Edge to Leading Edge (LELE) technique, similar to all national centres^19,20^. External and internal validation of the ultrasonographic examinations is performed annually^18^. A longer description can be found in the *supplementary methods* and an overview of the care trajectory for patients with an AAA is visualized in *Fig. S1*.

Men invited but not participating in the programme are defined as non-participants and men invited and participating in the ultrasonographic examination are defined as participants. Men with an aortic diameter of ≥30 mm are defined as having an AAA, men with an aortic diameter of 25–29 mm are defined as having a subaneurysmal aorta, and men with an aortic diameter of <17 mm are defined as having a small aorta, similar to previous definitions^21,22^.

Men who are diagnosed with an AAA before invitation to screening commonly cancel their screening appointment, as they are aware of their diagnosis; thus, they are not included in this cohort. If a man participates in the screening examination even though he has already been diagnosed with an AAA, he is not included in the screening database at the outpatient visit, as all previously diagnosed men are excluded from the screening database.

The data are presented according to the STROBE guidelines^23^.

### Ethical considerations

The study was approved by the Swedish Ethical Review Authority (reference number 2024-02389-01) and it did not require informed consent from the programme participants.

### Data analysis

#### Entire cohort of screened persons

Prevalence estimates were calculated among the participants and confidence intervals for prevalence estimates were calculated based on a binomial probability distribution. Descriptive statistics include means, medians, and truncated means, which exclude the highest and lowest 10% of values. Persons invited or examined in 2024 were excluded from the analysis.

For survival analysis of the entire cohort of screening participants, the screening registry did not provide an exact date of death for every participant. Deaths were only updated for participants who did not move from Stockholm and Gotland counties, so survival analysis for the entire cohort was limited to participants registered in these regions (112 780). For the patients with a diagnosed aneurysm at screening, the date of death was manually retrieved from electronic healthcare records and, in this case, this was used instead when analysing only patients with an AAA. To estimate the error in classification of date of death within the registry data, the presumed date of death from the registry was compared with the actual recorded date of death. The difference was >1 year for four patients (0.3%) (being 3200 days for 1 of these patients) and it was <60 days for the remaining patients.

To determine the effect of screening aortic diameter on mortality, two different models were used. The Kaplan–Meier method was used to estimate crude all-cause mortality. Cox proportional hazards regression was used to model the effect of aortic diameter on all-cause mortality, among persons with a normal aortic diameter. To allow for a non-linear relationship between mortality and aortic diameter, a restricted cubic spline was used. The lowest Akaike information criterion (AIC) was achieved for seven knots (compared with 3 and 5 knots and linear) and this is the reported association. Non-linearity was tested using the likelihood ratio test^24^. A proportional hazards assumption was tested using Schoenfeld residuals.

#### Cohort of patients with an AAA

The cohort of men diagnosed at screening with an AAA ≥30 mm were invited to an outpatient visit at the Department of Vascular Surgery. At the initial visit they filled out a questionnaire regarding co-morbidities, medication, heredity, and use of nicotine products. Based on the data collected from the questionnaire and clinical examination, the vascular surgeon or specialist nurse filled out a web-based form from the regional online screening programme regarding risk factors and planned surveillance or treatment.

In the cohort of patients diagnosed with an AAA, some patients had a subaneurysmal diameter at the subsequent ultrasonographic examination and surveillance was discontinued, these men were excluded from this analysis. The percentage of missing data was ≤2.5% for all risk factors, except for cigarettes per day, years smoking, and pack-years smoking, for which the data are presented in the patient characteristics, but not analysed further. Missing observations were excluded for other variables. The Kaplan–Meier method was used to estimate the crude incidence of all-cause mortality and the Aalen-Johansen estimator was used to estimate the crude cumulative incidence of surgical repair when accounting for the competing risk of death, implemented in R package *tidycmprsk*^25^. Adjusted all-cause mortality estimates were computed using flexible parametric survival regression and cause-specific cumulative incidence was computed^26,27^. The cumulative incidence of surgical repair was only estimated in patients with an aortic diameter of <50 mm at the initial visit. Adjusted models included and did not include AAA diameter at the initial visit, to investigate the total effect of the exposure (including the size at the initial visit) and the effect that was independent of the size at the initial visit. All patients were of the same age and sex, so these factors were not included in the models. Models were adjusted for factors that may influence AAA progression or diameter, that is smoking, diabetes, and statins^28,29^.

AAA growth rates depending on patient characteristics were modelled using mixed-effects models that were either linear or log-linear. Models either included only time (years), the modelled covariate, and their interaction (crude) or were also adjusted for maximal diameter at the initial visit and other specified factors, by including an interaction term with baseline diameter and time. All models included patient-specific random intercepts and slopes for time. The estimates that are presented are marginal trends for the relevant patient characteristics, and differences and *P* values are pairwise comparisons with the indicated reference level. R packages were used for fitting mixed-effects models (‘lme4’ and ‘lmerTest’) and for marginal means (‘marginaleffects’)^30–32^.

## Results

### Invited and participating men

During the study period (July 2010 to June 2024), 152 850 men, all 65 years of age, were invited to an aortic ultrasonographic examination (*Fig. S2*). The overall participation rate was 78%.

#### Prevalence and diameter

In total, 117 120 persons with a registered aortic measurement were included for further analysis. The mean(s.d.) infrarenal aortic diameter decreased during the study period, from 18.6(3.3) mm to 18.1(2.8) mm (*Table 1*). AAA prevalence during the entire study period was 1.00% (95% c.i. 0.95% to 1.06%), but declined during the study period, from 1.32% to 0.69% (*Table 1* and *Fig. S3*). A similar proportion of men had a subaneurysmal aorta during the study period compared with the proportion who had an AAA, which also showed a decreasing trend, from 1.53% (95% c.i. 1.40% to 1.67%) to 0.77% (95% c.i. 0.68% to 0.88%). The proportion of men with small aortas (<17 mm) was larger and varied between 16.43% (95% c.i. 16.03% to 16.83%) and 21.94% (95% c.i. 21.47% to 22.43%) during the study period.

**Table 1:** Infrarenal aortic diameter among the 117 120 persons who attended screening during 2010–2023

Time interval	Number of participants examined	Infrarenal aortic diameter (mm)			Prevalence (95% c.i.), %		
		Mean(s.d.)	Truncated mean	Median (i.q.r.)	≥30 mm	25–29 mm	<17 mm
2010–2013	31 307	18.59(3.32)	18.26	18 (17–20)	1.32 (1.20,1.45)	1.53 (1.40,1.67)	18.09 (17.67,18.53)
2014–2017	32 580	18.61(3.10)	18.32	18 (17–20)	1.08 (0.97,1.20)	1.11 (1.00,1.23)	16.43 (16.03,16.83)
2018–2020	24 608	18.43(2.85)	18.19	18 (17–19)	0.86 (0.75,0.98)	1.03 (0.91,1.16)	17.73 (17.26,18.22)
2020–2023	28 625	18.14(2.81)	17.93	18 (17–19)	0.69 (0.60,0.79)	0.77 (0.68,0.88)	21.94 (21.47,22.43)
Overall	117 120	18.45(3.05)	18.18	18 (17–19)	1.00 (0.95,1.06)	1.12 (1.06,1.18)	18.50 (18.27,18.72)

Confidence intervals for prevalence estimates were calculated based on a binomial probability distribution. A truncated mean excludes the highest and lowest 10% of values. i.q.r., interquartile range.

#### Mortality in men with small and large aortic diameters

**Fig. 1:**
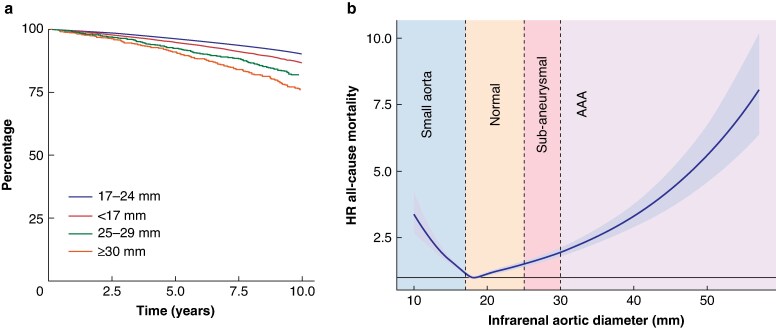
Mortality according to infrarenal aortic diameter at screening **a** Kaplan–Meier-estimated survival according to infrarenal aortic diameter at screening, for persons divided into four groups, as indicated by the labels. **b** Cox proportional hazards model of survival by aortic diameter at time of screening. Diameter was modelled using a restricted cubic spline and the estimated hazard function is shown. The reference was set at 18 mm (the median infrarenal aortic diameter), which corresponds to the dashed line. Analysis was limited to persons with diameters <60 mm. AAA, abdominal aortic aneurysm.

In a total of 112 780 examined individuals, 8798 deaths occurred during the total follow-up time of 753 789 person-years. Crude estimates of overall survival are displayed in *Fig. 1a* and *Table 2* (presented for the four different groups according to their initial aortic diameter). At 5 years, the mortality was lowest among the group with an infrarenal diameter of 17–24 mm (3.8% (95% c.i. 3.7% to 3.9%)). Individuals with smaller and larger infrarenal aortic diameters had a higher all-cause mortality. The mortality rate at 5 years for individuals with an infrarenal diameter <17 mm was 5.5% (95% c.i. 5.1% to 5.8%), among individuals with a subaneurysmal aorta (25–29 mm) it was 8.1% (95% c.i. 6.4% to 9.7%), and among individuals with an AAA; 9.5% (95% c.i. 7.6% to 11.3%) mortality rate. The risk ratio for death at 5 years was 1.44 (95% c.i. 1.34 to 1.56) for individuals with a small aorta at screening, 2.13 (95% c.i. 1.76 to 2.69) for individuals with subaneurysms, and 2.50 (95% c.i. 2.09 to 3.10) for individuals with an AAA (*Table 2*).

**Table 2:** Crude cumulative incidence of all-cause mortality (Kaplan–Meier estimate) according to size of infrarenal diameter at screening and relative risk ratios

	Normal (17–24 mm)	Small (<17 mm)	Subaneurysmal	AAA
**Follow-up**				
Persons, *n*	89 268	21 146	1244	1122
Deaths, *n*	6406	1966	197	229
Person-years	603 260	133 603	8980	7946
Duration (years), median (i.q.r.)	7.12 (2.85–10.63)	6.39 (2.59–10.41)	8.24 (4.71–11.59)	8.31 (4.78–11.46)
**5 years**				
All-cause mortality (95% c.i.), %	3.8 (3.7,3.9)	5.5 (5.1,5.8)	8.1 (6.4,9.7)	9.5 (7.6,11.3)
Difference, %	0 (reference)	1.7 (1.3,2.1)	4.3 (2.6,6.0)	5.7 (3.9,7.6)
Ratio	1 (reference)	1.44 (1.34,1.56)	2.13 (1.76,2.69)	2.50 (2.09,3.10)
**10 years**				
All-cause mortality (95% c.i.), %	10.5 (10.2,10.8)	14.2 (13.5,14.8)	19.4 (16.5,22.2)	26.1 (22.7,29.4)
Difference, %	0 (reference)	3.7 (2.9,4.4)	8.9 (6.2,11.8)	15.6 (12.3,19.1)
Ratio	1 (reference)	1.35 (1.28,1.43)	1.85 (1.62,2.18)	2.49 (2.21,2.88)

AAA, abdominal aortic aneurysm; i.q.r., interquartile range.

Using Cox proportional hazards regression, the association between aortic diameter and overall mortality deviated from linearity (*P* < 0.001 for non-linearity). Individuals with both small and large aortic diameters had increased HRs for all-cause mortality (*P* < 0.001) (*Fig. 1b*).

### Persons diagnosed with an AAA

In the 1174 patients with an AAA detected through the screening programme, 15 patients had a subaneurysmal diameter at some time during surveillance and were registered as non-AAA patients, and were therefore excluded from further analysis.

#### Temporal trends in risk factors and aneurysm progression

The distribution of AAA size at screening was similar in the early and late time intervals (*Table S1*). When the risk factor distribution among men with an AAA was compared between the early and late time intervals, only smoking habits and statin use differed. Patients in the late time interval were more often never smokers (15% *versus* 10%; *P* = 0.003), while a history of use of snus was more common (15% *versus* 12%; *P* = 0.021). Patients in the late time interval were also more likely to be treated with statins (49% *versus* 39%; *P* = 0.001).

**Fig. 2:**
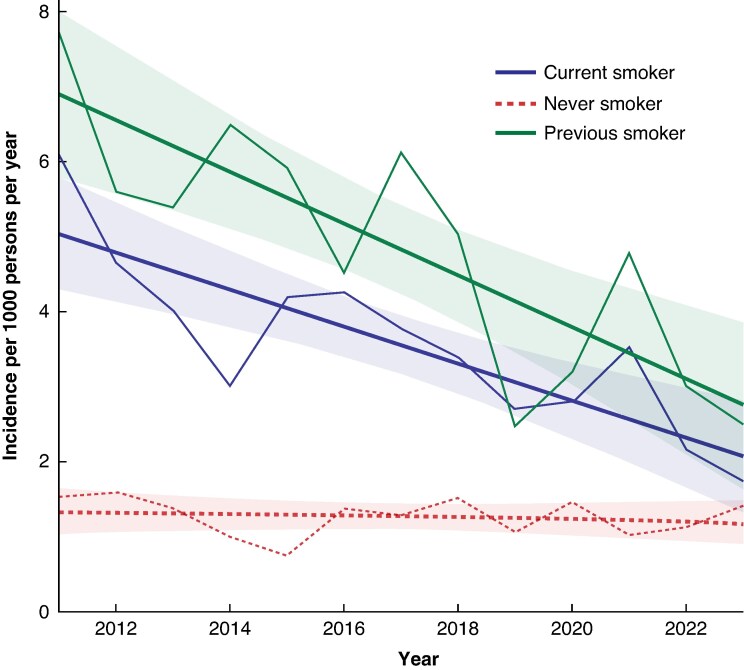
AAA incidence by smoking status at screening Incidence calculated for the screened population for each year. Lines represent the linear trends with 95% confidence intervals. AAA, abdominal aortic aneurysm.

A more detailed temporal analysis of smoking status showed that the yearly incidence of patients with an AAA reported as current and previous smokers decreased over the study period (the linear trend for the incidence of current smokers and previous smokers with an AAA was −0.25 (95% c.i. −0.35 to −0.14) per 1000 persons/year and −0.35 (95% c.i. −0.45 to −0.24) per 1000 persons/year respectively), whereas the incidence of patients with an AAA reported as never smokers did not change over the study period (−0.01 (95% c.i. −0.12 to 0.09) per 1000 persons/year) (*Fig. 2*).

Comparing the early and late time intervals there was no difference in overall survival or cumulative incidence of surgery (0.8% (95% c.i. −2.2% to 3.8%)) (*Fig. S4*) in crude or adjusted analysis (*Table S2*). There was no difference in the aneurysm growth rate between the AAAs diagnosed in the different time intervals (2.03 (95% c.i. 1.85 to 2.21) mm/year in the early time interval *versus* 2.15 (95% c.i. 1.91 to 2.39) mm/year in the late time interval; *P* = 0.445).

#### Effect of risk factors on aneurysm presentation, progression, and mortality

Risk factors that differed across the time intervals were investigated to determine the influence on overall survival, AAA size at presentation, AAA growth, and progression to surgery. AAA patients who were current smokers at the initial visit had an increased all-cause mortality in crude and in adjusted analysis; at 5 years, 11.1% (95% c.i. 8.8% to 13.9%), compared with 5.6% (95% c.i. 3.4% to 9.2%) for non-smokers and 8.6% (95% c.i. 6.8% to 10.7%) for previous smokers. Snus and statin use did not influence all-cause mortality, in either crude or adjusted analysis (*Tables S3*, *S4*).

#### Progression to surgery

**Fig. 3:**
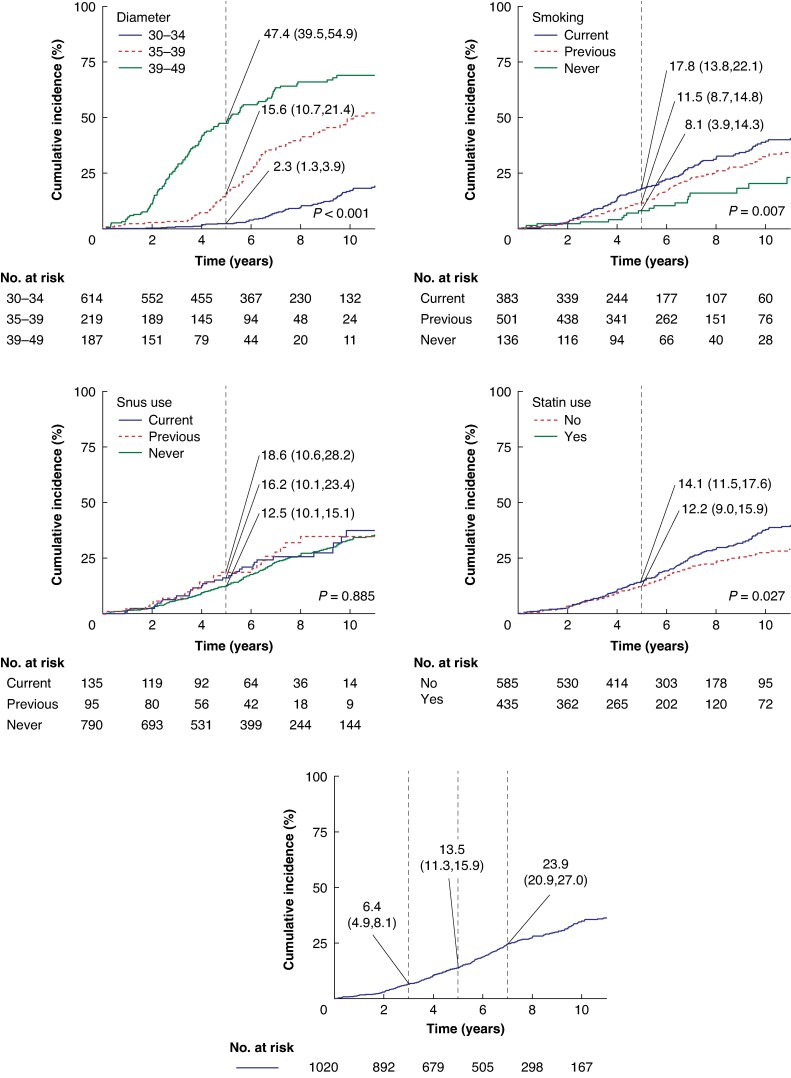
Crude estimates of cumulative incidence of surgery, overall and by index maximal diameter, smoking status, snus use, and statin use Limited to patients with an AAA and a maximal diameter of <50 mm at screening. AAA, abdominal aortic aneurysm.

For the overall cohort of patients with an AAA with a diameter of <50 mm, the crude incidence of surgical repair at 5 years was 14% (95% c.i. 11% to 16%) (*Fig. 3*). Both smoking and use of statins, but not snus, influenced the crude cumulative incidence of surgical repair (*Fig. 3* and *Table S3*). In adjusted analysis, smoking, but not use of statins, remained strongly associated with surgical repair. Specifically, only current smoking was associated with progression to surgery, both adjusted and unadjusted for baseline diameter, while previous smoking was only associated with progression to surgery in the model that was unadjusted for initial diameter (*Figs S5*, *S6* and *Tables S5*, *S6*). For use of snus, there was no association in the crude or the adjusted analysis.

#### Aneurysm size at screening

Patients who were current or previous smokers had larger aneurysms at the initial visit compared with patients who were non-smokers (mean diameters of 38.1 mm for current smokers and 38.0 mm for previous smokers compared with 35.2 mm for non-smokers; *P* = 0.003 and *P* = 0.003). Neither statin use nor snus use influenced baseline diameter (*Table S7*).

#### Growth rate

Smoking, but not the use of snus, influenced the AAA growth rate. Current smokers had a growth rate of 2.63 (95% c.i. 2.44 to 2.82) mm/year and never smokers had a growth rate of 1.54 (95% c.i. 1.21 to 1.86) mm/year. The AAA growth rate increased progressively with larger initial diameters, ranging from 1.47 (95% c.i. 1.35 to 1.58) mm/year for aneurysms measuring 30–35 mm to 3.36 (95% c.i. 2.81 to 3.90) mm/year for aneurysms measuring 45–50 mm. Additionally, individuals with diabetes exhibited a significantly lower aneurysm growth rate (1.70 (95% c.i. 1.39 to 2.00) mm/year) compared with non-diabetics (2.31 (95% c.i. 2.18 to 2.43) mm/year)) (*Table S8*).

### Risk factors associated with a small infrarenal aortic diameter

Risk factors associated with a small infrarenal aortic diameter were investigated in a subset of 226 persons with a normal aortic diameter and it was found that smoking habits may be associated with a small infrarenal aortic diameter; see *supplementary methods*, *supplementary results*, *Tables S9*, *S10*, and *Fig. S7*.

## Discussion

The decline in the prevalence rate of AAA and subaneurysmal aortas aligns with reported trends and is in contrast with the slight rise in the number of men with small aortas. The most alarming finding in this population-based contemporary screening cohort of 65-year-old men is the high mortality in all men with small or aneurysmal aortas, which is increased 1.5–2.5 times at 5 years, compared with men with normal aortic diameters. Although the increased mortality for persons with an AAA is well known, this finding is especially interesting to note for the almost one-fifth of the examined men with small aortic diameters, since this increased mortality rate affects an even larger group of persons in the population.

The strong beneficial influence of smoking cessation on the prolonged time to AAA surgery and survival supports intensified lifestyle modifications. The results also highlight the possible chance to identify a different phenotype; the subgroup of men who develop an AAA without having smoked—the incidence of men in this subgroup did not change, in contrast to the decline in the incidence of men with an AAA and a smoking history.

This population-based analysis of aortic diameters in the male population in general reveals that there is a parallel decline in aortic diameter among the aneurysmal and subaneurysmal groups, and in the mean aortic diameter in the population, which confirms other reports of decreasing AAA prevalence in the UK and Sweden^9,33–35^. The cohort is a group of 65-year-old men from a mixed urban and rural region participating in the ultrasonographic examination of their aortas. Their demographics mimic men participating in other screening programmes^33^. The population in the region has in general moved towards a more beneficial lifestyle, with declining numbers of smokers, increasing numbers of successful use of secondary prevention, and decreasing cardiovascular death rates during the observation interval^11,36^.

Interestingly, independently of the decline in the prevalence of AAAs (and subaneurysms), the normal diameter of the aorta is declining. Aortic diameter, independently of aneurysmal disease, is associated with ageing^37^, which is likely multifactorial, but related to the breakdown of elastin^38^, which is especially implicated in AAA, but also other conditions, with smoking as a strong risk factor.

The high all-cause mortality of patients with an AAA compared with the general population is known and has been reported to be 2–4 times higher than for matched men in the population^39^. The high mortality risk remains, regardless of age at treatment and treatment method, and is even higher in women than in men^40^ , which, unfortunately, could not be explored in the present study. The overall mortality rate is clearly stratified when aortic diameter from screening is used as a marker. This could have implications for future secondary prevention programmes and the use of screening programmes based on aortic ultrasonographic examination. As most screening programmes report only on diagnosed AAA patients, this is generally difficult to explore. In the present study there is a u-shaped effect on mortality, with increased mortality for small and large diameters. Although worse survival and an association with peripheral arterial disease have been reported previously among individuals with small aortic diameters, there is a lack of knowledge on the pathogenesis or cost-effectiveness for earlier identification of this patient group^15,16,22,41^. The results of the present study also indicate that smoking habits may be associated with small aortic diameters. Together, these results form a basis for future explorations, including translational studies.

The proportion of men diagnosed with an AAA is declining in Sweden, as well as in the UK screening programme, and patients diagnosed with an AAA only show small differences over time; the distribution of diameters, progression to surgery, and risk factors are mostly similar. The patient group only differs over time regarding the unchanged number of non-smokers with an AAA, in contrast to the declining trend of smokers with an AAA. The unchanged number of men presenting with an AAA, but with no history of smoking, stresses the need to treat this subgroup of men as a different phenotype. They present with smaller index diameters and have a slower progression of disease, but show an unchanged risk of developing disease, compared with the declining number of men with an AAA and a smoking history. Future stratgies could be to specifically focus on other phenotype risk-groups, other than men and smokers, with risk factors for AAA development; such as hyperlipidaemia, heredity, chronic kidney disease, or other hitherto unknown^42,43^. Snus use in this study was not associated with mortality, or aneurysm-related outcomes, which is in line with previous results where no relation to aneurysms or peripheral arterial disease has been observed^44,45^ .

A positive finding in the cohort of men with an AAA in the present study was the support of the exceptionally strong positive influence of smoking cessation on outcome, in harmony with prior reports^46,47^. Men who have stopped smoking have a larger index diameter than non-smokers, but their rates of AAA growth, progression to surgery, and mortality are better than those of the men who continue to smoke. These findings should support more intensified efforts for programmes targeting smokers.

The regional population-based screening programme with a 78% participation rate in the present study, carried out at very centralized high-flow ultrasonographic units, has generated a high-quality database. The Swedish National Board of Health and Welfare validated the screening programme in 2024, showing a declining national trend in AAA^8^. The non-participants (22% in the present study) presumably have a higher prevalence of AAA and a more pathological risk factor profile, but the proportion of small aortic diameters is unknown^20^. Unfortunately, women are not screened, but may be equally or better served by a screening cardiovascular risk stratification of small infrarenal aortic diameters. The risk factor analysis of small aortic diameters must be viewed as indicative due to the limited data.

The clearly decreasing number of men who develop aortic aneurysm follows a general decrease in aortic width in the population. The higher mortality risk in men with very small aortic diameters or larger aneurysmatic aortas is clearly shown in this contemporary report. This highlights that an identification of a target population for more intensified secondary prevention, in men with small aortic diameters could give a secondary beneficial effect by the screening programme. In this study, the devastating effects of smoking on rates of progression to surgery, AAA growth, and mortality are confirmed. The support for smoking cessation therapy in men with an AAA is clearly shown by the reduced rates of progression to surgery for non-smokers and previous smokers compared with current smokers.

## Supplementary Material

znaf156_Supplementary_Data

## Data Availability

Access to data from the Stockholm Abdominal Aortic Aneurysm screening registry may be applied for through the Regional Cancer Centrum in Stockholm after acquisition of relevant ethical permits.
