# Supplementary material for: Decreasing aortic diameter and decreasing prevalence of infrarenal aortic aneurysms in a population-based screening programme
Source: Br J Surg. 2025 Aug 20;112(8):znaf156. doi: 10.1093/bjs/znaf156 (PMC12366718; doi:10.1093/bjs/znaf156)
Supplement: znaf156_Supplementary_Data [file znaf156_supplementary_data.docx]

**Aortic Diameter and Prevalence of Infrarenal Aortic Aneurysms are Decreasing in a Population-Based Screening Programme**

Siika, Antti ^1^;Axelsson, Anton^1^; Fattahi, Nina ^1,2^; Roy Joy ^1,2^; Öhman, Daniel^3^; Linné, Anneli; Hultgren, Rebecka ^1,2^

**Affiliations:** ^1^Department of Molecular Medicine and Surgery, Karolinska Institutet, Stockholm, Sweden**,** ^2^ Department of Vascular Surgery, Karolinska Institutet and University Hospital, Stockholm, Sweden**,** ^3^ Regional Cancer Center Stockholm-Gotland, ^4^ Department of Clinical Science and Education, Karolinska Institutet at Södersjukhuset, ^5^ Department of Surgery, Section of Vascular Surgery, Södersjukhuset,Stockholm, Sweden.

**Corresponding Author:** Rebecka Hultgren**,** [rebecka.hultgren@ki.se](mailto:rebecka.hultgren@ki.se)**,** ME Kärlkirurgi, Norrbacka S3: 01, Karolinska Universitetssjukhuset, 171 76 Solna, Sweden**,** Telephone: +468-123 76596

**Supplementary Materials - Index**

[**Supplementary Methods** 3](#_Toc200986379)

[Description about the Stockholm Screening Program 3](#_Toc200986380)

[Cohort of persons with normal aortic diameter 4](#_Toc200986381)

[Statistical analysis of factors associated with small normal aortic diameter 4](#_Toc200986382)

[**Supplementary Results** 5](#_Toc200986383)

[Factors associated with small aortic diameter 5](#_Toc200986384)

[References 6](#_Toc200986385)

[**Supplementary Figures** 7](#_Toc200986386)

[**Supplementary Figure 1**. Care trajectory of patients with abdominal aortic aneurysm including detection and surveillance. Figure created with Biorender.com 7](#_Toc200986387)

[**Supplementary Figure 2.** Flow chart describing persons invited into the screening program, and detection of AAAs and sub-aneurysms. *Note that 2024 did not represent a complete year in the data set. Figure created with Biorender.com 8](#_Toc200986388)

[**Supplementary Figure 3.** Prevalence of abdominal aortic aneurysms and sub-aneurysms and small aorta (<17mm) per year, presented as proportion of examined population, with 95% confidence interval. 9](#_Toc200986389)

[**Supplementary Figure 4.** Cause-specific cumulative incidence of surgical repair by time period. Regression standardized estimates of (A) cumulative incidence of surgical repair for an early and late time period with 95 CI, and (B) difference between the early and late time period. Estimates are adjusted 10](#_Toc200986390)

[**Supplementary Figure 5.** Cumulative incidence of surgery with 95% Confidence Intervals according to smoking status. Regression standardized cause-specific estimates. (**A**) not adjusted for index diameter, and (**B**) adjusted for index diameter. Top panel shows cumulative incidence of surgery, and bottom panels show difference. 11](#_Toc200986391)

[**Supplementary Figure 6.** Cause-specific cumulative incidence of surgical repair according to statin use. (A) Not adjusted for baseline diameter, and (b) adjusted for baseline diameter. Adjusted for smoking. 12](#_Toc200986392)

[**Supplementary Figure 7. Relation between years smoked and infrarenal aortic diameter,** among persons with normal aortic diameter in the screening program. Blue lines show quantile regression estimates for the 5^th^ and 10^th^ percentiles. 13](#_Toc200986393)

[**Supplementary Tables** 14](#_Toc200986394)

[**Supplementary Table 1.** Aneurysm size and patient characteristics for 1,159 patients with abdominal aortic aneurysm divided by time-period. 14](#_Toc200986395)

[**Supplementary Table 2**. Crude estimate of all-cause mortality and cumulative incidence of surgical repair for patients in an early (2010-2016) and a late (2016-2023) time period. %, 95% CI. 16](#_Toc200986396)

[**Supplementary Table 3.** Crude estimates of all-cause mortality and cumulative incidence of surgical repair (% with 95% CI), according to smoking, snus and statin status at index. 17](#_Toc200986397)

[**Supplementary Table 4.** Regression standardized estimates of **all-cause mortality** according to smoking, use of Swedish snus and statins (% with 95 CI%). 18](#_Toc200986398)

[**Supplementary Table 5.** Cause-specific regression standardized estimates of **cumulative incidence of surgical repair** (%, 95% CI) according to smoking status, snus status and statin use. Estimates in patients that had a less than 50 mm aneurysm at screening. 19](#_Toc200986399)

[**Supplementary Table 6.**  Cause-specific regression standardized estimates of **cumulative incidence of surgical repair** (%, with 95% CI) according to smoking status, snus status and statin use. **Adjusted also for index diameter.** 20](#_Toc200986400)

[**Supplementary Table 7.** Difference Initial diameter at screening for patients with AAA by smoking status, Swedish snus status, and use of statin 21](#_Toc200986401)

[**Supplementary Table 8.** Difference in diameter growth rate for patients with AAA by Initital diameter, smoking status, Swedish snus status, use of statin and diabetes. 22](#_Toc200986402)

[**Supplementary Table 9.** Cohort of 226 persons with normal infrarenal aortic diameter 23](#_Toc200986403)

[**Supplementary Table 10.** Interval censored quantile regression, displaying factors associated with the 5^th^ and 10^th^ percentiles of infrarenal aortic diameter. 25](#_Toc200986404)

# **Supplementary Methods**

### Description about the Stockholm Screening Program

The coordination of the screening program is centralized to the Stockholm-Gotland Regional Cancer Center (RCC). A regional web-based database is used to coordinate the screening process and monitor and evaluate outcomes (invitations, reminders, ultrasound-based diameter of the aorta, risk factors and long-term surveillance results). Invitations are sent by regular mail, with a pre-scheduled appointment at an ultrasound clinic. Non-participants receive 2 reminders. Between 2010-2012 there was an administration fee; approximately 12€, after this date it became free of charge. This had no impact on participation-rate.^16^

The men diagnosed at the core ultrasound lab are referred within two weeks to the vascular surgery departments’ outpatient clinics. This is called the index visit. Surveillance regimes for AAA are based on regional protocols, harmonized within the National Swedish Screening group, SASS. (SSVS.nu/screening/SASS) The surveillance interval is determined by the latest measured maximum aneurysm diameter in accordance with international guidelines.^2,19^ Men diagnosed in the program with AAA are surveilled according to national and international recommendations. Men with SAA are not surveilled in this region, which is in accordance with the recommendations from the National Board of Health and Welfare.

*Definitions*

The web-based program holds patient demographics which are collected prospectively starting at the first visit if diagnosed with an AAA (*index*). During the subsequent surveillance visits, maximal aortic diameter measurements, possible aortic intervention, discontinued surveillance and death are registered. Study exposures registered at index are; age, sex, height, weight, smoking status (current, previous or never, pack years), high blood pressure (pharmacologically treated), angina, acute myocardial infarction, heart failure (pharmacologically treated), chronic pulmonary disease (COPD, defined as pharmacologically treated), renal insufficiency, malignancy, diabetes mellitus (managed with diet or pharmacologically), and any other relevant disease. Ongoing medication use at index was collected and grouped as antihypertensives, antiplatelets, warfarin, other anticoagulants, statins, and other lipid lowering drugs.

### Cohort of persons with normal aortic diameter

A cohort of persons with normal aortic diameter, previously recruited and described in a previous publication Villard et al. [1], were included to specifically study factors associated with a small infrarenal aortic diameter. In short, 226 individuals who underwent screening and had an infrarenal aortic diameter < 30 mm were randomly asked to participate in a questionnaire between January 1^st^ 2014 -December 31^st^ 2014.

### Statistical analysis of factors associated with small normal aortic diameter

Quantile regression was used to describe how the 5^th^ and 10^th^ percentiles varied with the characterized risk factors. As the ultrasound measurements of the infrarenal aortic diameter were only available as integer rounded data, an interval censored quantile regression, as implemented in the r-package *ctqr* [2], was used, where the upper and lower limits of the measurements were assumed to be .5 above and below the nominal measurement.

# **Supplementary Results**

### Factors associated with small aortic diameter

Quantile regression analysis showed that smoking status was associated with infrarenal renal aortic diameter, the 10^th^ and 5^th^ percentiles were estimated at -1.29 mm and -1.53 mm for current smokers compared to never smokers (p = 0.006 and 0.004, respectively). The 5^th^ percentile for previous smokers was also smaller (-1.08 mm, p = 0.025). Years smoked (Supplementary Figure 6), and pack-years were also associated with a decreased diameter at the 5^th^ and 10^th^ percentile (Supplementary Table 12).

Significant associations with decreased diameter were also found for hypertension, at the 5^th^ percentile and heart failure and kidney disease at the 5^th^ and 10^th^ percentiles. BMI was on the contrary associated with an increased diameter at the 5^th^ and 10^th^ percentiles (Supplementary Table 12).

### References

[1] C. Villard, J. Roy, M. Bogdanovic, P. Eriksson, and R. Hultgren, “Sex hormones in men with abdominal aortic aneurysm,” *J. Vasc. Surg.*, vol. 74, no. 6, pp. 2023–2029, Dec. 2021, doi: 10.1016/J.JVS.2021.06.020.

[2] P. Frumento, “A quantile regression estimator for interval-censored data,” *Int. J. Biostat.*, vol. 19, no. 1, pp. 81–96, May 2023, doi: 10.1515/ijb-2021-0063.

# **Supplementary Figures**


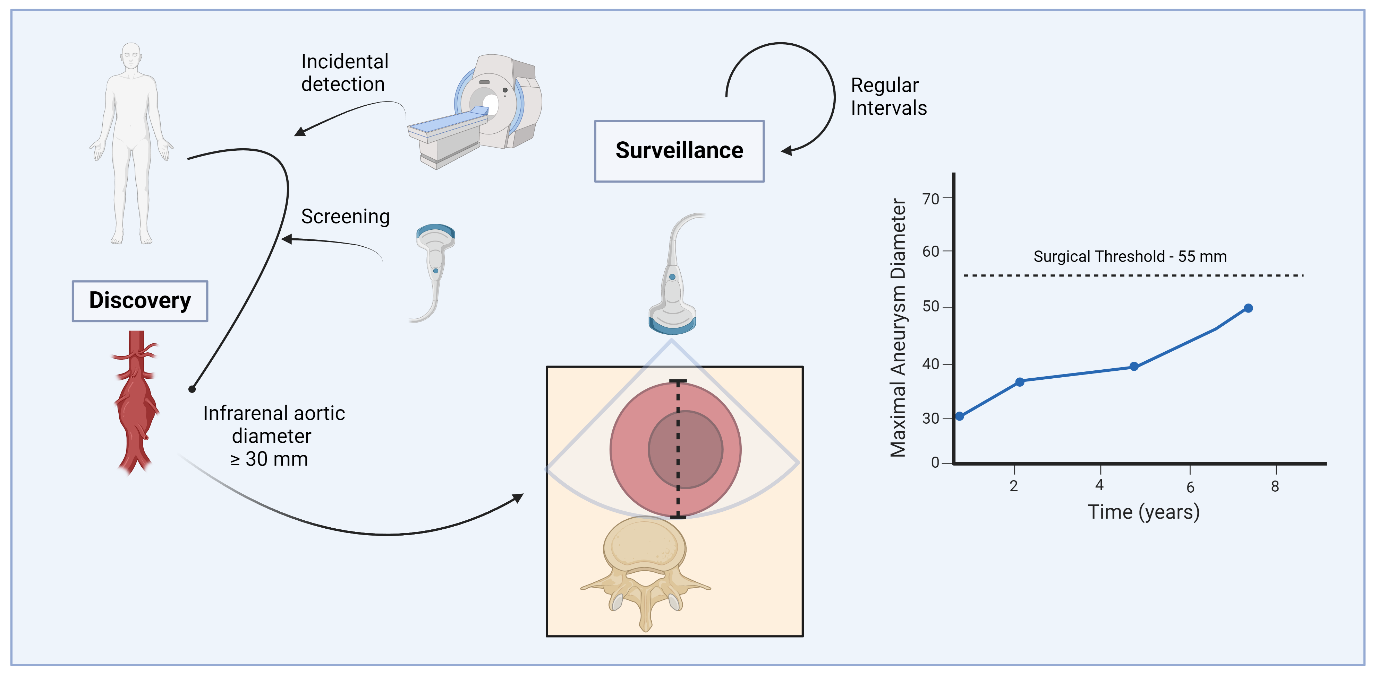


### **Supplementary Figure 1**. Care trajectory of patients with abdominal aortic aneurysm including detection and surveillance. Figure created with Biorender.com


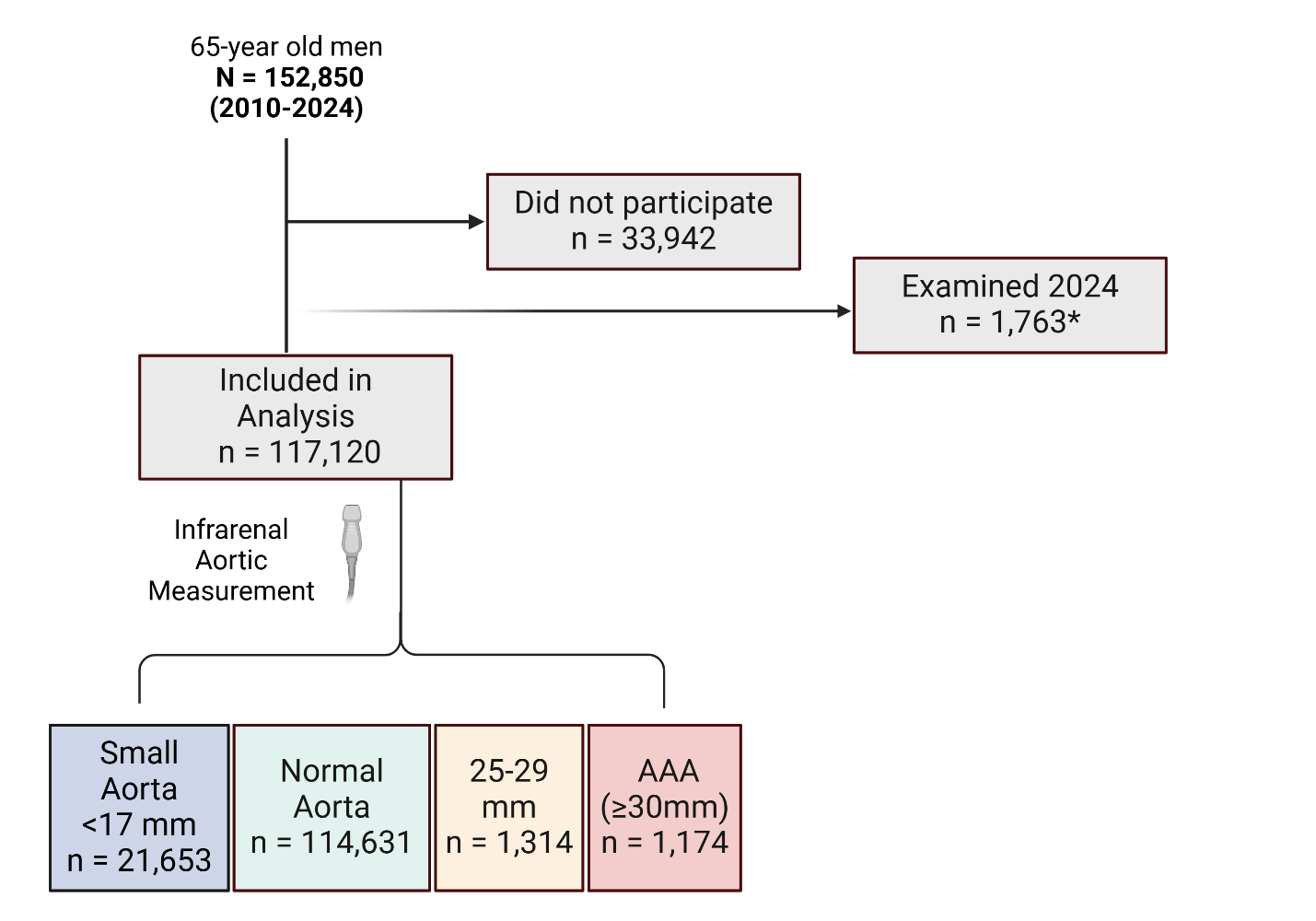


### **Supplementary Figure 2.** Flow chart describing persons invited into the screening program, and detection of AAAs and sub-aneurysms. *Note that 2024 did not represent a complete year in the data set. Figure created with Biorender.com


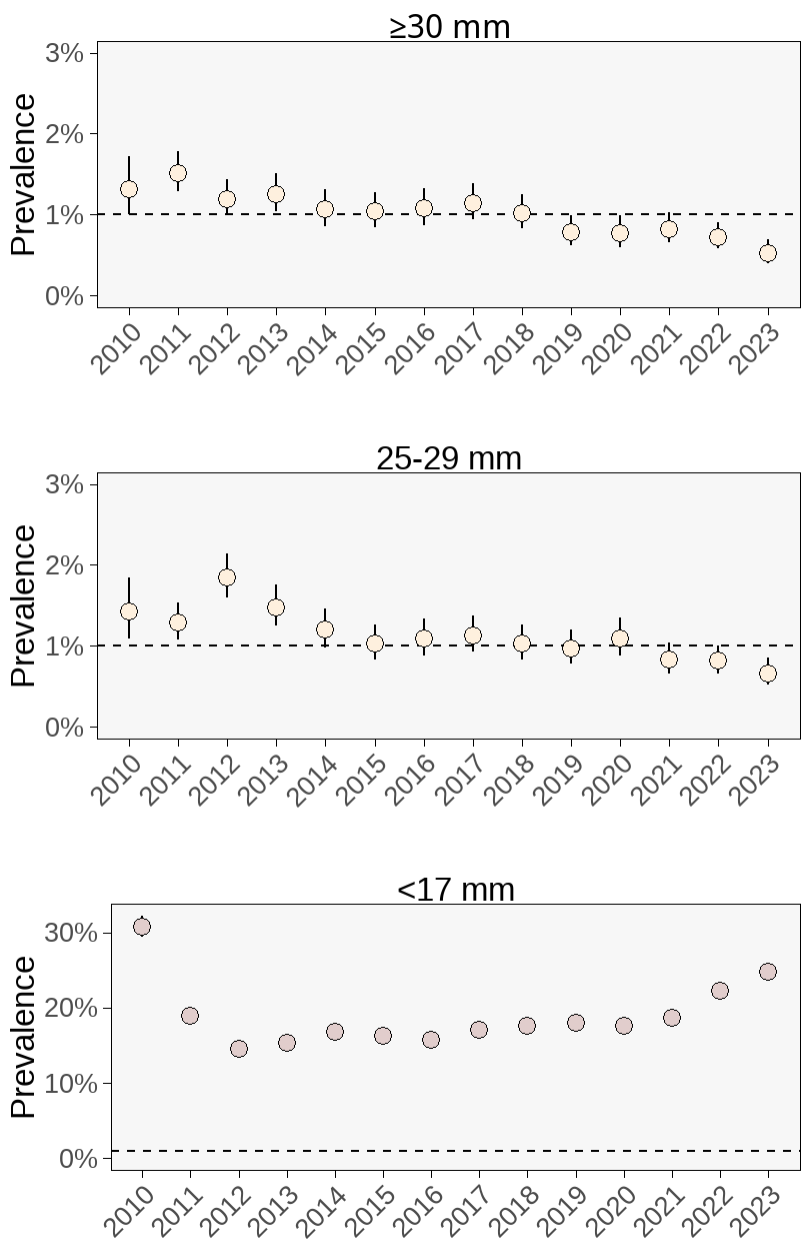


### **Supplementary Figure 3.** Prevalence of abdominal aortic aneurysms and sub-aneurysms and small aorta (<17mm) per year, presented as proportion of examined population, with 95% confidence interval.


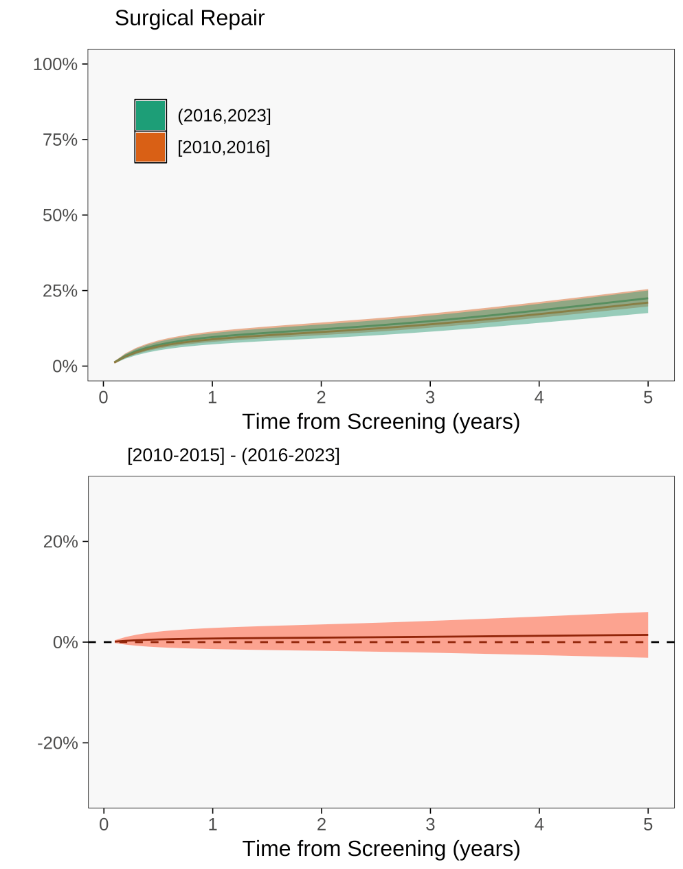


### **Supplementary Figure 4.** Cause-specific cumulative incidence of surgical repair by time period. Regression standardized estimates of (A) cumulative incidence of surgical repair for an early and late time period with 95 CI, and (B) difference between the early and late time period. Estimates are adjusted


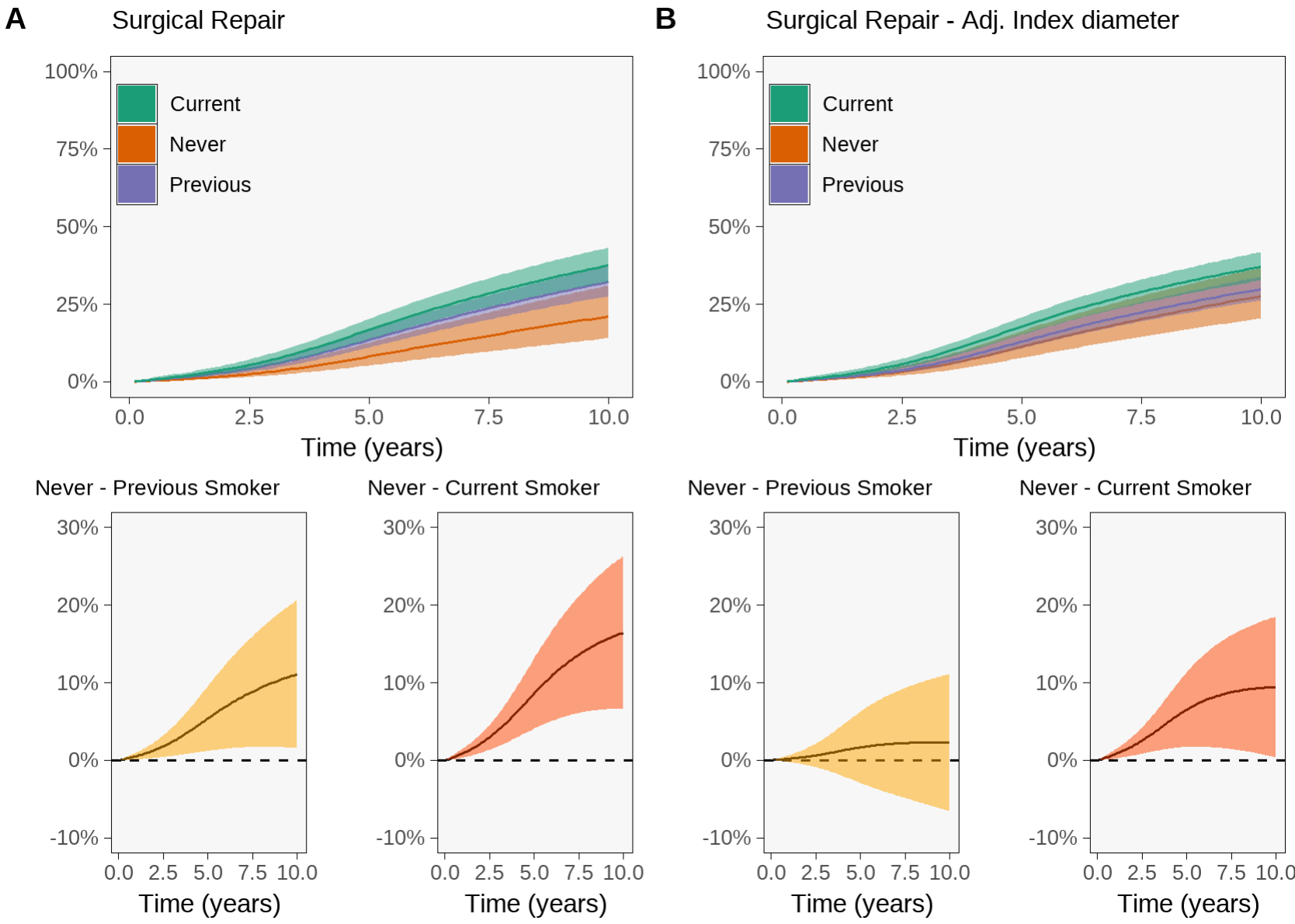


### **Supplementary Figure 5.** Cumulative incidence of surgery with 95% Confidence Intervals according to smoking status. Regression standardized cause-specific estimates. (**A**) not adjusted for index diameter, and (**B**) adjusted for index diameter. Top panel shows cumulative incidence of surgery, and bottom panels show difference.


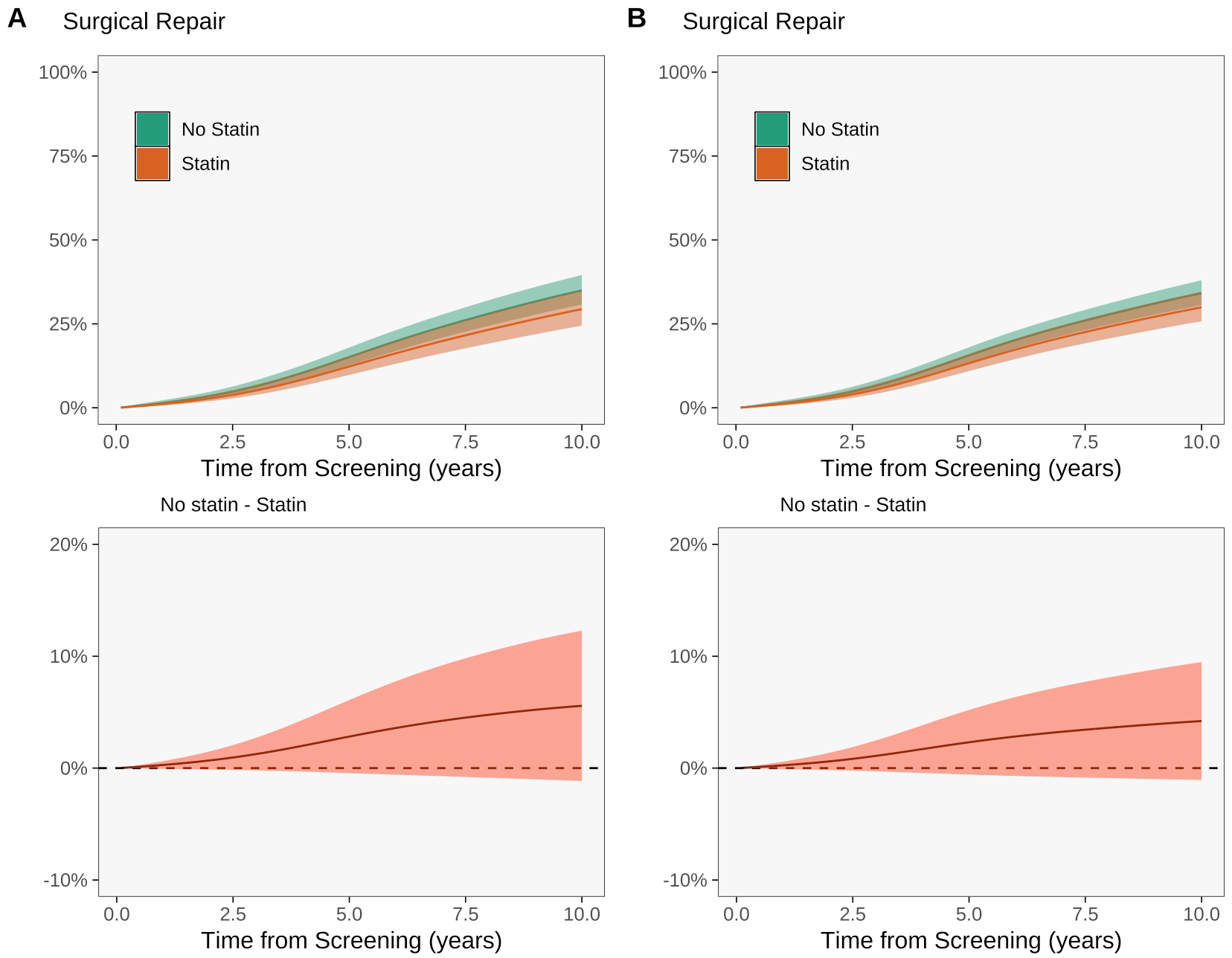


### **Supplementary Figure 6.** Cause-specific cumulative incidence of surgical repair according to statin use. (A) Not adjusted for baseline diameter, and (b) adjusted for baseline diameter. Adjusted for smoking.


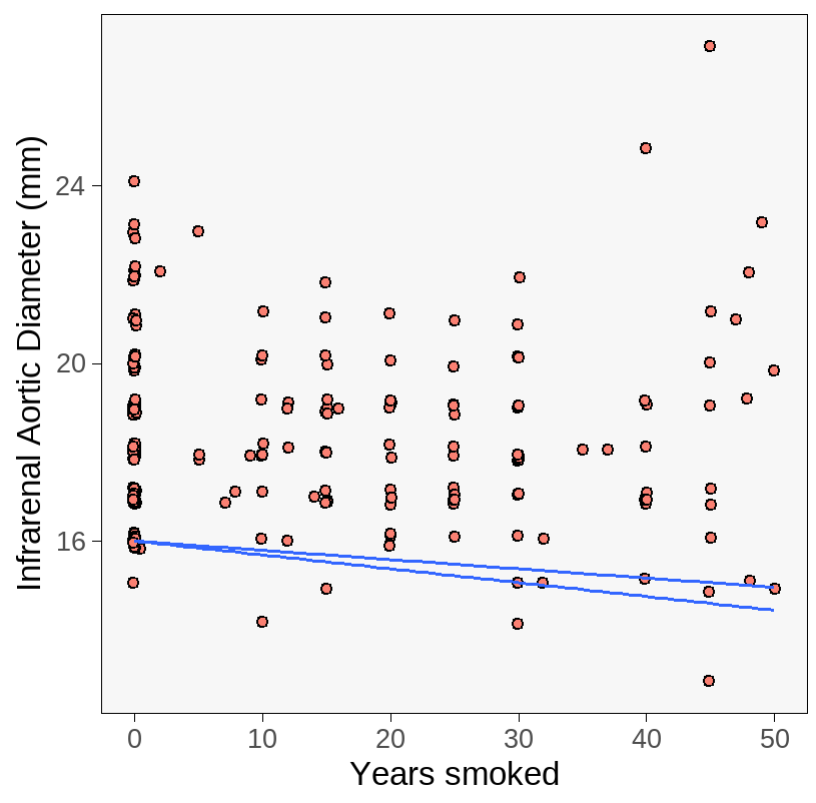


### **Supplementary Figure 7. Relation between years smoked and infrarenal aortic diameter,** among persons with normal aortic diameter in the screening program. Blue lines show quantile regression estimates for the 5^th^ and 10^th^ percentiles.

# **Supplementary Tables**

| **Supplementary Table 1.** Aneurysm size and patient characteristics for 1,159 patients with abdominal aortic aneurysm divided by time-period. | | | | |
| --- | --- | --- | --- | --- |
|  | **Overall** N = 1,159*^1^* | **2010-2016** N = 656*^1^* | **2016-2023** N = 503*^1^* | **p-value***^2^* |
| Maximal diameter (mm) | 34 (31 – 40) | 34 (31 – 41) | 34 (31 – 40) | 0·43 |
| Maximal diameter (categories) |  |  |  | 0·83 |
| <35 | 664 (57%) | 375 (57%) | 289 (57%) |  |
| (35,40] | 207 (18%) | 111 (17%) | 96 (19%) |  |
| (40,45] | 111 (9·6%) | 66 (10%) | 45 (8·9%) |  |
| (45,50] | 58 (5·0%) | 34 (5·2%) | 24 (4·8%) |  |
| >50 | 119 (10%) | 70 (11%) | 49 (9·7%) |  |
| Weight (kg) | 89 (80 – 100) | 89 (80 – 98) | 90 (80 – 100) | 0·34 |
| Height (cm) | 180 (175 – 184) | 180 (175 – 184) | 180 (175 – 183) | 0·29 |
| Smoking |  |  |  | **0·033** |
| Never | 142 (13%) | 67 (10%) | 75 (15%) |  |
| Current | 427 (38%) | 255 (40%) | 172 (35%) |  |
| Previous | 565 (50%) | 322 (50%) | 243 (50%) |  |
| Swedish snuff |  |  |  | **0·021** |
| Never | 879 (78%) | 518 (80%) | 361 (74%) |  |
| Previous | 102 (9·0%) | 48 (7·5%) | 54 (11%) |  |
| Current | 153 (13%) | 78 (12%) | 75 (15%) |  |
| Cigarettes per day | 15 (10 – 20) | 15 (10 – 20) | 15 (10 – 20) | **0·029** |
| Years smoking | 40 (25 – 45) | 40 (25 – 45) | 35 (22 – 45) | 0·11 |
| Pack-years smoking | 25 (15 – 40) | 29 (17 – 40) | 25 (13 – 40) | **0·0090** |
| Angina Pectoris | 118 (10%) | 74 (11%) | 44 (9·0%) | 0·17 |
| Previous MI | 215 (19%) | 128 (20%) | 87 (18%) | 0·37 |
| Heart Faliure | 46 (4·1%) | 23 (3·6%) | 23 (4·7%) | 0·34 |
| Hypertension | 652 (57%) | 366 (57%) | 286 (58%) | 0·60 |
| Lung disease | 122 (11%) | 68 (11%) | 54 (11%) | 0·80 |
| Diabetes | 170 (15%) | 86 (13%) | 84 (17%) | 0·077 |
| Kidney function impairment | 35 (3·1%) | 15 (2·3%) | 20 (4·1%) | 0·091 |
| Malignancy | 36 (3·2%) | 24 (3·7%) | 12 (2·4%) | 0·22 |
| Other relevant disease | 137 (12%) | 82 (13%) | 55 (11%) | 0·44 |
| First degree relative | 110 (9·7%) | 64 (9·9%) | 46 (9·4%) | 0·76 |
| Beta-blocker | 368 (32%) | 210 (33%) | 158 (32%) | 0·90 |
| Warfarin | 47 (4·1%) | 27 (4·2%) | 20 (4·1%) | 0·93 |
| Other anticoagulant | 52 (4·6%) | 12 (1·9%) | 40 (8·2%) | **<0·0001** |
| Statin | 488 (43%) | 250 (39%) | 238 (49%) | **0·0010** |
| Other lipid lowering agent | 40 (3·5%) | 16 (2·5%) | 24 (4·9%) | **0·029** |
| *^1^*Median (IQR); n (%) | | | | |
| *^2^*Wilcoxon rank sum test; Pearson's Chi-squared test | | | | |

| **Supplementary Table 2**. Crude estimate of all-cause mortality and cumulative incidence of surgical repair for patients in an early (2010-2016) and a late (2016-2023) time period. %, 95% CI. | | | | |
| --- | --- | --- | --- | --- |
|  | 1 year | 3 years | 5 years | p-value*^1^* |
| **Crude** | | | | |
| All-cause mortality | | | | |
| Early [2010,2016] | 1·1 (0·3 - 1·8) | 5·3 (3·6 - 7·0) | 9·7 (7·4 - 12) |  |
| Late (2016,2023] | 1·3 (0·3 - 2·2) | 5·0 (2·8 - 7·2) | 8·0 (4·9 - 11) |  |
| Cumulative Incidence of Surgical repair | | | | |
| Early [2010,2016] | 1·5 (0·77 - 2·8) | 6·7 (4·9 - 8·9) | 13 (10 - 16) |  |
| Late (2016,2023] | 1·2 (0·45 - 2·6) | 5·8 (3·7 - 8·6) | 15 (11 - 19) |  |
| **Regression Standardized** |  |  |  |  |
| All-cause mortality |  |  |  |  |
| [2010,2016] | 1.3 (0.8 - 2.1) | 5.0 (3.7 - 6.6) | 9.5 (7.6 - 11.7) |  |
| (2016,2023] | 1.2 (0.7 - 2.0) | 4.5 (3.2 - 6.4) | 8.6 (6.2 - 11.7) |  |
| *(2016,2023] - [2010,2016]* | -0.1 (-0.6 - 0.4) | -0.5 (-2.3 - 1.3) | -0.9 (-4.2 - 2.4) |  |
| Cumulative Incidence of Surgical repair | | | | |
| [2010,2016] | 1.4 ( 0.8 - 2.2) | 6.2 ( 4.7 - 8.0) | 14.5 (12.1 - 17.3) |  |
| (2016,2023] | 1.2 ( 0.7 - 2.0) | 5.5 ( 4.0 - 7.6) | 13.2 (10.0 - 17.2) |  |
| *(2016,2023] - [2010,2016]* | 0.1 (-0.3 - 0.6) | 0.6 (-1.3 - 2.5) | 1.3 (-2.9 - 5.5) |  |
| *^1^*Log-rank test, or Gray’s Test. Cumulative incidence of surgical repair is estimated allowing for competing risk of death. Regression standardized estimates adjusted for smoking status, statin, diabetes and baseline diameter. | | | | |

| **Supplementary Table 3.** Crude estimates of all-cause mortality and cumulative incidence of surgical repair (% with 95% CI), according to smoking, snus and statin status at index. | | | | | |
| --- | --- | --- | --- | --- | --- |
|  | **1 Year** | **3 Years** | **5 Years** | **10 Years** | **p-value***^1^* |
| **All-cause mortality** | | | | | |
| Smoker |  |  |  |  | **0·012** |
| Never | 1·4 (0 - 3·3) | 3·0 (<0·1 - 5·9) | 5·9 (1·5 - 10) | 13 (6·0 - 20) |  |
| Current | 0·9 (<0·1 - 1·8) | 6·0 (3·6 - 8·2) | 11 (7·9 - 14) | 31 (25 - 37) |  |
| Previous | 1·2 (0·3 - 2·1) | 5·2 (3·2 - 7·0) | 8·5 (6·0 - 11) | 25 (20 - 29) |  |
| Snus |  |  |  |  | 0·80 |
| Never | 1·2 (0·5 - 2·0) | 5·8 (4·2 - 7·4) | 9·4 (7·3 - 11) | 26 (22 - 30) |  |
| Previous | 0 (0 - 0) | 2·5 (0 - 5·9) | 9·1 (2·4 - 15) | 33 (18 - 46) |  |
| Current | 1·3 (0 - 3·1) | 3·4 (0·4 - 6·2) | 8·2 (3·4 - 13) | 22 (13 - 30) |  |
| Statin |  |  |  |  | 0·94 |
| No Statin | 1·1 (0·3 - 1·9) | 5·1 (3·3 - 6·8) | 9·5 (7·1 - 12) | 26 (21 - 30) |  |
| Statin | 1·2 (0·2 - 2·2) | 5·3 (3·2 - 7·4) | 8·6 (5·9 - 11) | 27 (21 - 32) |  |
| **Cumulative Incidence of Surgery** | | | | | |
| Smoker |  |  |  |  | **0·007** |
| Never | 2·3 (0·61 - 6·0) | 3·1 (1·0 - 7·3) | 8·1 (3·9 - 14) | 20 (12 - 30) |  |
| Current | 1·1 (0·36 - 2·5) | 8·2 (5·6 - 11) | 18 (14 - 22) | 39 (33 - 45) |  |
| Previous | 1·4 (0·63 - 2·8) | 5·8 (3·9 - 8·2) | 12 (8·7 - 15) | 32 (27 - 38) |  |
| Snus |  |  |  |  | 0·9 |
| Never | 1·4 (0·75 - 2·4) | 6·0 (4·4 - 7·9) | 12 (10 - 15) | 33 (29 - 38) |  |
| Previous | 2·2 (0·41 - 6·9) | 7·0 (2·8 - 14) | 19 (11 - 28) | 35 (23 - 47) |  |
| Current | 0·77 (0·07 - 3·9) | 8·1 (4·1 - 14) | 16 (10 - 23) | 37 (26 - 49) |  |
| Statin |  |  |  |  | **0·027** |
| No Statin | 1·6 (0·77 - 2·8) | 6·5 (4·7 - 8·8) | 14 (12 - 18) | 38 (33 - 43) |  |
| Statin | 1·2 (0·45 - 2·6) | 6·2 (4·1 - 8·9) | 12 (9·0 - 16) | 27 (22 - 33) |  |
| *^1^*Log-rank test, Gray’s test. Cumulative Incidence of Surgery is estimated allowing for competing risk of death. Cumulative Incidence of Surgery is estimated in patients that had a less than 50 mm aneurysm at screening. | | | | | |

| **Supplementary Table 4.** Regression standardized estimates of **all-cause mortality** according to smoking, use of Swedish snus and statins (% with 95 CI%). | | | | |
| --- | --- | --- | --- | --- |
|  | 1 Year | 3 Years | 5 Years | 10 Years |
| **Smoking** | | | | |
| Never | 0.8 (0.4 - 1.5) | 2.9 (1.7 - 4.9) | 5.6 (3.4 - 9.2) | 16.8 (10.6 - 26.0) |
| Current | 1.5 (0.9 - 2.5) | 5.8 (4.4 - 7.7) | 11.1 (8.8 - 13.9) | 31.0 (26.3 - 36.5) |
| Previous | 1.2 (0.7 - 1.9) | 4.5 (3.4 - 5.9) | 8.6 (6.8 - 10.7) | 24.7 (20.8 - 29.2) |
| Difference Current – Never | 0.8 (0.2 - 1.3) | 2.9 (1.0 - 4.8) | 5.4 (2.0 - 8.9) | 14.2 (5.3 - 23.2) |
| Difference Previous- Never | 0.4 (-0.1 - 0.9) | 1.5 (-0.1 - 3.2) | 2.9 (-0.2 - 6.1) | 7.9 (-0.6 - 16.4) |
| **Swedish snus** | | | | |
| Never | 1.3 (0.8 - 2.0) | 4.9 (3.8 - 6.3) | 9.4 (7.7 - 11.4) | 26.8 (23.5 - 30.6) |
| Previous | 1.1 (0.6 - 2.2) | 4.4 (2.6 - 7.2) | 8.4 (5.2 - 13.4) | 24.2 (15.8 - 36.1) |
| Current | 1.1 (0.6 - 2.0) | 4.4 (2.9 - 6.7) | 8.4 (5.7 - 12.4) | 24.3 (17.2 - 33.7) |
| Difference Previous – Never | -0.1 ( -0.7 - 0.4) | -0.5 ( -2.7 - 1.6) | -1.0 ( -5.0 - 3.0) | -2.6 (-13.1 - 7.9) |
| Difference Current - Never | -0.1 ( -0.6 - 0.3) | -0.5 ( -2.3 - 1.3) | -1.0 ( -4.3 - 2.4) | -2.5 (-11.2 - 6.2) |
| **Statin** | | | | |
| No Statin | 1.3 (0.8 - 2.0) | 4.9 (3.7 - 6.4) | 9.3 (7.5 - 11.5) | 26.5 (22.7 - 30.7) |
| Statin | 1.2 (0.7 - 2.0) | 4.7 (3.5 - 6.3) | 9.0 (7.1 - 11.4) | 25.6 (21.3 - 30.7) |
| Difference Statin – No statin | 0.0 (-0.4 - 0.3) | -0.2 (-1.4 - 1.1) | -0.3 (-2.7 - 2.0) | -0.8 (-6.8 - 5.1) |

| **Supplementary Table 5.** Cause-specific regression standardized estimates of **cumulative incidence of surgical repair** (%, 95% CI) according to smoking status, snus status and statin use. Estimates in patients that had a less than 50 mm aneurysm at screening. | | | | |
| --- | --- | --- | --- | --- |
|  | 1 Year | 3 Years | 5 Years | 10 Years |
| **Smoking** | | | | |
| Never | 0.7 (0.4 - 1.3) | 3.3 (2.0 - 5.4) | 8.1 (5.2 - 12.5) | 21.0 (14.0 - 31.0) |
| Previous | 1.2 (0.8 - 2.0) | 5.6 (4.3 - 7.4) | 13.4 (11.0 - 16.4) | 32.1 (27.6 - 37.2) |
| Current | 1.6 (1.0 - 2.5) | 7.1 (5.4 - 9.3) | 16.7 (13.7 - 20.2) | 37.5 (32.3 - 43.2) |
| Previous – Never | 0.5 (0.1 - 1.0) | 2.3 (0.5 - 4.2) | 5.4 (1.2 - 9.5) | 11.1 (1.5 - 20.6) |
| Current - Never | 0.9 (0.3 - 1.4) | 3.8 (1.8 - 5.9) | 8.6 (4.0 - 13.1) | 16.4 (6.6 - 26.3) |
| **Snuff** | | | | |
| Never | 1.3 (0.8 - 2.0) | 5.7 (4.5 - 7.3) | 13.6 (11.5 - 16.1) | 32.1 (28.5 - 36.1) |
| Current | 1.4 (0.8 - 2.4) | 6.2 (4.2 - 9.1) | 14.5 (10.4 - 20.1) | 33.7 (25.3 - 43.8) |
| Previous | 1.5 (0.8 - 2.7) | 6.8 (4.3 - 10.6) | 15.9 (10.6 - 23.3) | 36.2 (25.7 - 49.2) |
| Current – Never | 0.1 (-0.4 - 0.6) | 0.4 (-1.8 - 2.6) | 0.9 (-4.0 - 5.8) | 1.5 (-8.3 - 11.4) |
| Previous – Never | 0.2 (-0.4 - 0.9) | 1.0 (-1.8 - 3.9) | 2.2 (-4.1 - 8.6) | 4.0 (-8.2 - 16.3) |
| **Statin** | | | | |
| No statin | 1.4 (0.9 - 2.3) | 6.4 (5.0 - 8.2) | 15.1 (12.7 - 17.9) | 35.0 (30.7 - 39.6) |
| Statin | 1.1 (0.7 - 1.8) | 5.1 (3.8 - 6.9) | 12.3 (9.8 - 15.4) | 29.4 (24.4 - 35.1) |
| Statin- No statin | 0.3 (-0.1 - 0.6) | 1.3 (-0.2 - 2.7) | 2.8 (-0.4 - 6.1) | 5.6 (-1.1 - 12.3) |

| **Supplementary Table 6.**  Cause-specific regression standardized estimates of **cumulative incidence of surgical repair** (%, with 95% CI) according to smoking status, snus status and statin use. **Adjusted also for index diameter.** | | | | |
| --- | --- | --- | --- | --- |
|  | 1 Year | 3 Years | 5 Years | 10 Years |
| **Smoking** | | | | |
| Never | 0.9 (0.5 - 1.6) | 4.3 (2.7 - 6.9) | 11.2 (7.6 - 16.3) | 27.6 (20.4 - 36.7) |
| Previous | 1.1 (0.7 - 1.7) | 5.1 (4.0 - 6.6) | 12.9 (10.8 - 15.3) | 29.8 (26.2 - 33.9) |
| Current | 1.6 (1.0 - 2.6) | 7.6 (6.0 - 9.6) | 17.7 (15.1 - 20.8) | 37.0 (32.6 - 41.8) |
| Previous – Never | 0.2 (-0.3 - 0.6) | 0.8 (-1.2 - 2.8) | 1.7 (-2.9 - 6.2) | 2.3 (-6.6 - 11.1) |
| Current - Never | 0.8 (0.2 - 1.3) | 3.3 (1.0 - 5.5) | 6.5 (1.7 - 11.3) | 9.4 (0.3 - 18.5) |
| **Snuff** | | | | |
| Never | 1.3 (0.8 - 2.0) | 6.1 (4.9 - 7.6) | 14.7 (12.7 - 16.9) | 32.5 (29.4 - 35.9) |
| Current | 1.1 (0.6 - 2.0) | 5.4 (3.7 - 7.8) | 13.2 (9.9 - 17.7) | 29.7 (23.4 - 37.4) |
| Previous | 1.4 (0.8 - 2.6) | 6.6 (4.4 - 9.9) | 15.7 (11.3 - 21.5) | 33.9 (25.8 - 43.6) |
| Current – Never | -0.2 ( -0.6 - 0.3) | -0.7 ( -2.6 - 1.2) | -1.4 ( -5.5 - 2.6) | -2.8 (-10.2 - 4.6) |
| Previous – Never | 0.1 (-0.5 - 0.7) | 0.5 (-2.1 - 3.1) | 1.0 (-4.2 - 6.2) | 1.3 (-7.9 - 10.6) |
| **Statin** | | | | |
| No statin | 1.4 (0.9 - 2.2) | 6.5 (5.2 - 8.2) | 15.6 (13.4 - 18.0) | 34.1 (30.6 - 38.0) |
| Statin | 1.1 (0.7 - 1.8) | 5.4 (4.1 - 7.1) | 13.3 (10.9 - 16.1) | 29.9 (25.8 - 34.6) |
| Statin- No statin | 0.2 (-0.1 - 0.6) | 1.1 (-0.3 - 2.5) | 2.3 (-0.6 - 5.2) | 4.2 (-1.1 - 9.5) |

| **Supplementary Table 7.** Difference Initial diameter at screening for patients with AAA by smoking status, Swedish snus status, and use of statin | | |
| --- | --- | --- |
|  | Index Diameter* | |
| **Smoking** | mm (95% CI) | P-value |
| Never | 35.2 (33.6, 36.9) | - |
| Previous | 38.0 (37.2, 38.9) | 0·003 |
| Current | 38.1 (37.2, 39.1) | 0·003 |
| **Swedish Snuff** |  |  |
| Never | 37.9 (37.2, 38.5) | - |
| Previous | 37.3 (35.2, 39.5) | 0·538 |
| Current | 37.9 (36.1, 39.6) | 0·302 |
| **Statin** |  |  |
| No | 37.7 (36.9, 38.5) | - |
| Statin | 37.7 (36.8, 38.6) | 0·239 |
| *Models for snus and statin are adjusted for Smoking status | |  |

| **Supplementary Table 8.** Difference in diameter growth rate for patients with AAA by Initital diameter, smoking status, Swedish snus status, use of statin and diabetes. | | | | |
| --- | --- | --- | --- | --- |
|  | Growth (Crude) | | Growth (Adjusted) | |
|  | mm/year (95% CI) | P-value | mm/year (95% CI) | P-value |
| **Initial Diameter (mm)** | | | | |
| 30-35 | 1.47 (1.35, 1.58) | - | 1.13 (0.97, 1.28) | - |
| 35-40 | 2.46 (2.24, 2.68) | **<0·001** | 2.08 (1.83, 2.32) | **<0·001** |
| 40-45 | 2.87 (2.56, 3.17) | **<0·001** | 2.57 (2.25, 2.88) | **<0·001** |
| 45-50 | 3.36 (2.81, 3.90) | **<0·001** | 2.96 (2.42, 3.51) | **<0·001** |
| **Smoking** | | | | |
| Never | 1.54 (1.21, 1.86) | - | 1.40 (1.11, 1.69) | - |
| Previous | 2.08 (1.92, 2.24) | **0·042** | 1.72 (1.56, 1.89) | **0·010** |
| Current | 2.63 (2.44, 2.82) | **<0·001** | 2.19 (2.00, 2.38) | **<0·001** |
| **Swedish Snuff** | | | | |
| Never | 2.27 (2.13, 2.40) | - | 1.87 (1.71, 2.03) | - |
| Prev. | 2.06 (1.67, 2.46) | 0·656 | 1.78 (1.43, 2.13) | 0·523 |
| Current | 2.10 (1.78, 2.42) | 0·620 | 1.77 (1.48, 2.06) | 0·590 |
| **Statin** | | | | |
| No | 2.31 (2.16, 2.47) | - | 1.85 (1.67, 2.03) | - |
| Statin | 2.08 (1.89, 2.26) | 0·051 | 1.69 (1.50, 1.87) | **0·018** |
| **Diabetes** | | | | |
| No | 2.31 (2.18, 2.43) | - | 2.12 (2.10, 2.32) | - |
| Yes | 1.70 (1.39, 2.00) | **<0·001** | 1.42 (1.24, 1.76) | **<0·001** |
| 'Mixed-effects model estimates, with patient-specific random slopes and intercepts, either crude or adjusted for baseline diameter, smoking, statin and diabetes. Difference represents the comparison between each category and the reference, and the p-value corresponds to a test of that difference. For variables than Intial diameter, adjusted estimates are shown at a reference diameter of 35mm. | | | | |

| **Supplementary Table 9.** Cohort of 226 persons with normal infrarenal aortic diameter | |
| --- | --- |
|  | **N = 226***^1^* |
| Infrarenal aortic diameter | 18·00 (17·00 – 19·00) |
| Height | 180·0 (176·0 – 184·0) |
| Unknown | 5 |
| Weight | 85 (78 – 95) |
| Unknown | 7 |
| BMI | 26·1 (24·1 – 28·4) |
| Unknown | 12 |
| Smoking |  |
| Never | 111 (49) |
| Previous | 87 (38) |
| Current | 28 (12) |
| No. Cigarettes per day | 0 (0 – 15) |
| Unknown | 26 |
| Years smoked | 0 (0 – 25) |
| Unknown | 4 |
| Pack years smoked | 0 (0 – 16) |
| Unknown | 27 |
| Swedish snus |  |
| Never | 179 (80) |
| Previous | 30 (13) |
| Current | 16 (7·1) |
| Unknown | 1 |
| Heredity | 18 (8·2) |
| Unknown | 6 |
| Angina | 8 (3·6) |
| Unknown | 2 |
| AMI | 17 (7·5) |
| Heart Failure | 5 (2·2) |
| Hypertension | 102 (45) |
| Lung disease | 12 (5·3) |
| diabetes | 18 (8·0) |
| Unknown | 1 |
| Kidney Disease | 7 (3·1) |
| Unknown | 1 |
| ASA | 21 (9·4) |
| Unknown | 2 |
| Statin use | 39 (18) |
| Unknown | 6 |
| Beta-blockers | 25 (12) |
| Unknown | 25 |
| ACE inhibitors | 33 (16) |
| Unknown | 25 |
| Ca inhibitors | 24 (12) |
| Unknown | 24 |
| Ang II-inhibitors | 20 (10·0) |
| Unknown | 25 |
| *^1^*Median (IQR); n () | |

| **Supplementary Table 10.** Interval censored quantile regression, displaying factors associated with the 5^th^ and 10^th^ percentiles of infrarenal aortic diameter. | | | | |
| --- | --- | --- | --- | --- |
|  | **10th percentile** | | **5^th^ percentile** | |
|  | **Estimate** | **P-value** | **Estimate** | **P-value** |
| Height | 0.02 | 0.434 | 0.04 | 0.286 |
| Weight | 0.01 | 0.723 | -0.01 | 0.581 |
| BMI | 0.01 | **<0.001** | 0.01 | **<0.001** |
| Smoking Previous | -0.26 | 0.208 | -1.08 | **0.025** |
| Smoking Current | -1.29 | **0.006** | -1.53 | **0.004** |
| No. Cigarettes per day | -0.02 | 0.330 | -0.07 | **0.001** |
| Years smoked | -0.03 | **<0.001** | -0.04 | **<0.001** |
| Pack-years smoking | -0.03 | **<0.001** | -0.04 | **<0.001** |
| Swedish Snus | 0.15 | 0.415 | 0.43 | 0.058 |
| Heredity | -0.84 | 0.280 | -0.93 | 0.109 |
| Angina | 0.19 | 0.662 | 0.72 | 0.126 |
| AMI | -0.08 | 0.856 | -1.29 | 0.459 |
| Heart Faliure | -2.25 | **<0.001** | -2.08 | **<0.001** |
| Hypertension | -0.20 | 0.336 | -0.94 | 0.032 |
| Lung disease | -0.04 | 0.870 | 0.63 | 0.164 |
| Diabetes | -0.86 | 0.266 | -1.01 | 0.075 |
| Kidney Disease | -2.05 | **0.002** | -1.93 | **0.006** |
| ASA | -0.06 | 0.849 | 0.12 | 0.800 |
| Statin use | -0.20 | 0.583 | -1.05 | 0.093 |
| Beta-blockers | -0.19 | 0.687 | -1.06 | 0.546 |
| ACEi | -0.42 | 0.542 | -0.96 | **0.006** |
| Ca inhibitors | 0.02 | 0.954 | 0.52 | 0.396 |
| Ang II-inhibitor | -0.72 | 0.495 | -0.90 | 0.142 |
|  | | | | |
